# Identification and Physicochemical Characterization of a New Allergen from *Ascaris lumbricoides*

**DOI:** 10.3390/ijms21249761

**Published:** 2020-12-21

**Authors:** Velky Ahumada, María Manotas, Josefina Zakzuk, Lorenz Aglas, Sandra Coronado, Peter Briza, Peter Lackner, Ronald Regino, Galber Araujo, Fatima Ferreira, Luis Caraballo

**Affiliations:** 1 Institute for Immunological Research, University of Cartagena, Cartagena 130014, Colombia; vahumadac@unicartagena.edu.co (V.A.); manotas.mcarolina@javeriana.edu.co (M.M.); jzakzuks@unicartagena.edu.co (J.Z.); scoronador@unicartagena.edu.co (S.C.); rreginol@unicartagena.edu.co (R.R.); 2 Department of Biosciences, University of Salzburg, 5020 Salzburg, Austria; lorenz.aglas@sbg.ac.at (L.A.); Peter.Briza@sbg.ac.at (P.B.); peter.lackner@sbg.ac.at (P.L.); galber.rodriguesaraujo@sbg.ac.at (G.A.); fatima.ferreira@sbg.ac.at (F.F.)

**Keywords:** *Ascaris*, allergen, IgE, Asc l 5, cDNA-library

## Abstract

To analyze the impact of *Ascaris lumbricoides* infection on the pathogenesis and diagnosis of allergic diseases, new allergens should be identified. We report the identification of a new *Ascaris lumbricoides* allergen, Asc l 5. The aim of this study was to evaluate the physicochemical and immunological features of the Asc l 5 allergen. We constructed an *A. lumbricoides* cDNA library and Asc l 5 was identified by immunoscreening. After purification, rAsc l 5 was physicochemically characterized. Evaluation of its allergenic activity included determination of Immunoglobulin E (IgE) binding frequency (in two populations: 254 children and 298 all-age subjects), CD203c based-basophil activation tests (BAT) and a passive cutaneous anaphylaxis (PCA) mouse model. We found by amino acid sequence analysis that Asc l 5 belongs to the SXP/RAL-2 protein family of nematodes. rAsc l 5 is a monomeric protein with an alpha-helical folding. IgE sensitization to rAsc l 5 was around 52% in general population; positive BAT rate was 60%. rAsc l 5 induced specific IgE production in mice and a positive PCA reaction. These results show that Asc l 5 has structural and immunological characteristics to be considered as a new allergen from *A. lumbricoides*.

## 1. Introduction

The prevalence of allergic diseases has increased worldwide during the last few decades, probably depending on environmental and genetic influences. In the tropics, where allergic diseases and intestinal helminthiases are frequent [1,2], their relationships can influence the pathogenesis of both disorders. *Ascaris lumbricoides* infection (ascariasis) exerts a dual effect on allergic diseases such as asthma: it can increase prevalence and symptoms or diminish the allergic inflammation through immunomodulating molecules [3]. The immunostimulant effect of ascariasis has been explained by non-specific boosting of T helper 2 (Th2) immunity, direct effects of larvae migration [1,4] and cross-reactivity with house dust mite (HDM) allergens [5,6]. In addition, the existence of helminthiases has limited the use of traditional diagnostic markers of allergy (such as total and specific IgE) in the tropics, supporting the need for the detection of specific immune response. For those reasons, research on helminth/allergy interactions is of great scientific and clinical interest in both industrialized and developing countries [7].

So far, three allergens from *Ascaris* have been described, one species-specific (Asc s 1) and two cross-reactive with HDM (Asc l 3 and Asc l 13) [6,8,9,10]; however, the whole extract has at least nine additional IgE binding components [11]. Since *Ascaris* induces strong IgE responses even from an early age [12], other antigens could be potential allergens.

On the process of characterizing new allergens, it has been realized that allergenicity (IgE induction and binding capacity) is not enough to demonstrate the whole allergenic activity; other tests that confirm the involvement of candidate molecules in the inflammatory allergic reaction are necessary [13]. Furthermore, the physicochemical characterization of the molecule will help to understand both, biological and immunological properties. In connection with our previous work about *A. lumbricoides* allergen characterization [6,8], in the present work we constructed an *A. lumbricoides* cDNA library, isolated and expressed a new IgE-binding molecule (Asc l 5), identified its native counterpart in a natural source, analyzed its physicochemical attributes with a focus on aggregation behavior, ligand binding properties, secondary structural elements and bioinformatics analysis, and investigated the IgE-binding frequency and allergenic activity induced by Asc l 5 in humans and in a mouse model of a passive cutaneous anaphylaxis (PCA). As a result, we identified and characterized a new *A. lumbricoides* allergen.

## 2. Results

### 2.1. Asc l 5 Belongs to the SXP/RAL-2 Protein Family

**Figure 1:**
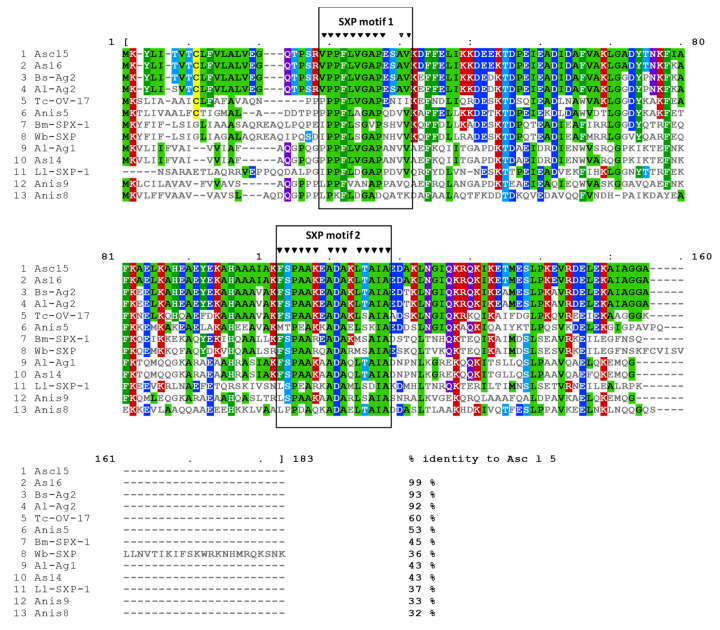
Amino acid sequence alignment of Asc l 5 with other members of the SXP/RAL-2 protein family. Each protein sequence is preceded by its name. Database entry code as follow: As16 from *Ascaris suum*: BAC66614.1. Ag2 from *Baylisascaris schroederi*: ACJ03762.1. Ag2 from *Ascaris lumbricoides*: ADB45852.1. OV-17 antigen from *Toxocara canis*: KHN84076.1. Ani s 5 from *Anisakis simplex*: BAF43534.1. SPX-1 from *Brugia malayi*: AAA27864.1. SXP antigen from *Wuchereria bancrofti*: AAC70783.1. Ag1 from *Ascaris lumbricoides*: ACJ03764.1. As14 from *Ascaris suum*: BAB67769.1. SXP-1 protein from *Loa loa*: AAG09181.1. Ani s 9 from *Anisakis simplex*: ABV55106.1. Ani s 8 from *Anisakis simplex*: BAF75681.1. SXP motif 1 and SXP motif 2 are shown (SXP motif 1 ([I:V]PPFLXGAPXXVV) and SXP motif 2 ([F:L]SP[E:A]A[R:K]XADAX-[M:L][S:T]AIA).

Immunoscreening of the *A. lumbricoides* cDNA expression library with patients’ sera detected three IgE-positive spots. The 3 clones were coding the same sequence that was submitted to the GenBank database under accession number MN275230; the allergen was designated as Asc l 5 by the International Union of Immunological Societies (IUIS) Nomenclature Committee. Sequence analysis showed that Asc l 5 belongs to SXP/RAL-2 protein family. SXP motif 1 and SXP motif 2 are shown in the sequence of Asc l 5 (Figure 1). When compared with sequences in databases, similarities were found with other nematode proteins, for example As16 from *Ascaris suum*, Ag2 from *Baylisascaris schroederi*, As14 from *A. suum,* and nematode infection markers such as SPX antigens from *Brugia malayi* and *Wuchereria bancrofti*. It was of great interest for us that there were three *Anisakis simplex* allergens in the same family: Ani s 5, Ani s 8 and Ani s 9. Since Ani s 5 is the only member of this protein family that has been physicochemically characterized and its 3D structure resolved by nuclear magnetic resonance (NMR, Protein Data Bank (PDB) entry 2MAR) [14], and considering the percent of identity between the two proteins (53%), it was very helpful for addressing the physicochemical characterization of Asc l 5.

### 2.2. Natural Asc l 5 in the A. lumbricoides Extract

**Figure 2:**
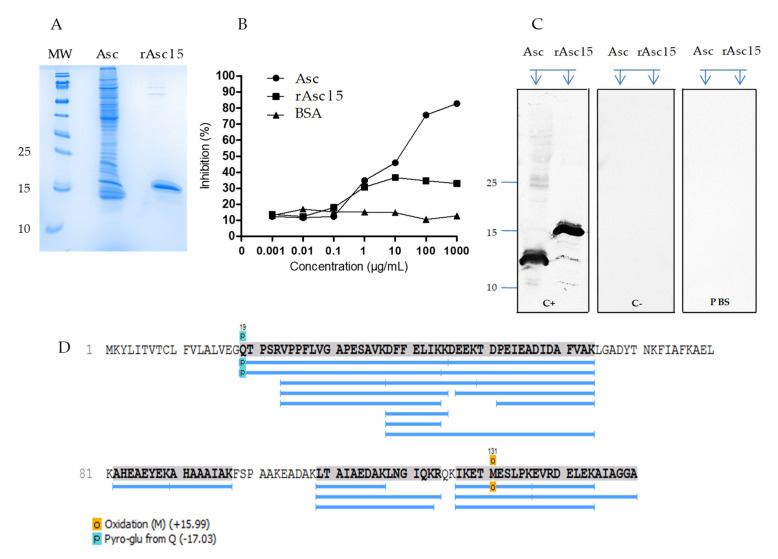
rAsc l 5 and its natural counterpart in *Ascaris lumbricoides* extract. (**A**) Sodium dodecyl sulphate-polyacrylamide gel electrophoresis (SDS-PAGE) of *A. lumbricoides* extract (5 µg) and rAsc l 5 (1 µg). (**B**) Enzyme-linked immunosorbent assay (ELISA) inhibition using a pool of sera with positive IgE to *Ascaris*; *A. lumbricoides* extract in solid phase. (**C**) Western blot probed with sera from mice immunized with rAsc l 5+Alum (C+) or phosphate-buffered saline (PBS) + Alum (C−) and PBS (as buffer control for non-specific binding of rat AP-conjugated anti-mouse IgG1 antibody) shows that natural Asc l 5 (nAsc l 5) is recognized in the *A. lumbricoides extract* (Lane Asc) and rAsc l 5 is recognized as well. Asc: *A. lumbricoides* extract. (**D**) Mass spectrometric analysis of peptide coverage of nAsc l 5. Blue lines indicate peptides identified by tandem mass spectrometry (MS/MS) from the *A. lumbricoides extract* matching mature sequence of Asc l 5. Amino acid sequence of Asc l 5 deposited in GenBank under accession number MN275230 is shown. Amino acids from 1 to 18 correspond to the signal peptide, mature sequence of Asc l 5 starts in amino acid number 19.

Mature Asc l 5 was expressed in *E. coli* origami (DE3) as a hexa-histidine-tagged protein. Under sodium dodecyl sulphate-polyacrylamide gel electrophoresis (SDS-PAGE, reduced conditions), rAsc l 5 migrated at a molecular mass of ∼16 KDa (Figure 2A); this result agreed with theoretical molecular weight of tagged recombinant of 16.9 kDa. Approximately 25 milligrams of the recombinant protein were obtained from 1 L of the expression medium. rAsc l 5 was purified to homogeneity, as determined by SDS-PAGE and Coomassie staining.

The purified rAsc l 5 inhibited around 30% of the IgE-binding to the *A. lumbricoides* extract, as determined in enzyme-linked immunosorbent assay (ELISA) inhibition (Figure 2B). Also, sera pool from *Ascaris*-positive IgE individuals recognized rAsc l 5 by WB (Figure S1). As expected, sera pool from infected subjects detected several bands in the *A. lumbricoides* extract, including a band close to ∼14KDa.

In order to have an accurate identification of natural Asc l 5 and to further evaluate its identity with the recombinant allergen, we used sera from mice immunized with rAsc l 5 to identify the natural protein (nAsc l 5) in the extract (Figure 2C, lane for Asc). In the WB, mice polyclonal sera recognized one band with a molecular weight of ∼14 kDa in the *A. lumbricoides* extract (Figure 2C), in agreement with the theoretical molecular weight of the mature nAsc l 5. The amino acid sequence of nAsc l 5 was confirmed by mass spectrometry performed using the in-solution trypsin-digested *A. lumbricoides* extract (Figure 2D and Table S2). High coverage (77%) of the protein sequence was obtained. In Figure 2D the amino acid sequence of nAsc l 5 is shown. The blue lines indicate the peptides identified by tandem mass spectrometry (MS/MS) sequencing.

### 2.3. rAsc l 5 Is a Monomeric Protein

**Figure 3:**
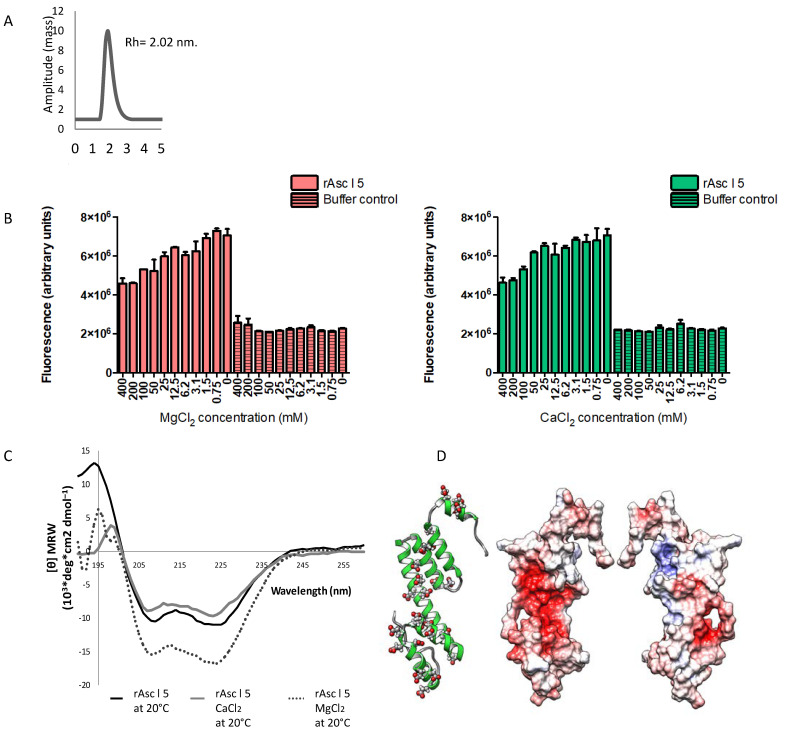
Physicochemical properties of recombinant Asc l 5. (**A**) Hydrodynamic radius (Rh) determined by dynamic light scattering (DLS). (**B**) 8-anilino-1-naphthalenesulfonic acid (ANS) assay, concentration dependent reduction of ANS binding to rAsc l 5 preincubated with different concentrations of Ca^2+^ or Mg^2+^. (**C**) Circular dichroism spectra of rAsc l 5 alone (100 µg/mL) or in presence of CaCl_2_ (74 mM CaCl_2_) or MgCl_2_ (221 mM) measured at 20 °C. (**D**) Homology model of Asc l 5 based on Ani s 5 structure (2MAR_A). Left side shows a ribbon representation of Asc l 5. Center and right side show electrostatic surface of Asc l 5. Negative charges are in red, positive charges are in blue. Right side, 180u rotated view.

rAsc l 5 was analyzed according to mass distribution of the protein in solution in DLS experiments showing that it has only one peak at 17.41 kDa in close agreement with the theoretical mass value of the hexa-histidine-tagged recombinant (16.9 kDa) and revealed a hydrodynamic radius (Rh) of around 2.02 nm. This indicates that rAsc l 5 is a monomer (Figure 3A). rAsc l 5 does not form aggregates, a feature also described for Ani s 5 [14]. High concordance was found between theoretical and experimental amino acid composition of rAsc l 5, confirming the identity of the recombinant and its concentration (950 µg/mL). The content of alpha helical structures was determined by Fourier transform infrared (FTIR) spectroscopy, being 52.8%.

### 2.4. Calcium and Magnesium Induce Structural Changes in rAsc l 5

Given the sequence similarity between Ani s 5 and Asc l 5, we explored if rAsc l 5 was able to bind Ca^2+^ or Mg^2+^. Garcia-Mayoral et al. [14], showed that the Ani s 5 structure resembles a Calmodulin-like fold. During Ani s 5′s physicochemical characterization, its ability to bind Ca^2+^ and Mg^2+^ was evaluated [14]. Also, we investigated if these ligands induced structural changes in Asc l 5, as has been described for Calmodulin, where remarkably changes in individual domains are observed after Ca^2+^ binding [15]. As a first approach, we studied rAsc l 5 by ANS experiments. ANS is a probe that increases its fluorescence upon binding to proteins (ANS alone has low fluorescence properties). ANS also has been used to correlate changes in fluorescence intensity with changes in protein tertiary structure [16]. If a natural ligand binds to the protein, less contact surface could be available for ANS binding and an expected dose-dependent reduction of the signal can be observed, closely related with changes in protein structure. As shown in Figure 3B, there were changes in ANS fluorescence when Ca^2+^ or Mg^2+^ were added to the protein preparation, detected as a dose-dependent decrease of the fluorescence signal, which is concordant with changes in protein structure and displacement of ANS by metal ions, suggesting that rAsc l 5 binds both Ca^2+^ and Mg^2+^. To further evaluate possible structural changes in rAsc l 5 induced by Ca^2+^ or Mg^2+^ we performed circular dichroism (CD) spectroscopy experiments. CD spectra confirmed that there were changes in rAsc l 5′s secondary structural elements induced by the addition of Ca^2+^ or Mg^2+^ (Figure 3C). Protein preparations used in CD assay were run in SDS-PAGE gel, as shown in Figure S2. Structural changes in rAsc l 5 are consistently demonstrated by CD and ANS fluorescence data, and might be necessary for interaction between Asc l 5 and calcium/magnesium.

### 2.5. Three-Dimensional Structure Model of Asc l 5 Comprises Six Alpha Helices with Negatively Charged Patches and Predicted Calcium and Magnesium-Binding Sites

The 3D structure model of Asc l 5 comprises six alpha helices as shown in ribbon representation of Figure 3D (left side). Electrostatic surface representations of Asc l 5 are also shown (center and right side). Negative and positive charges, as well as hydrophobic patches, are represented in the structure. Negative patches occupy most of a cavity depicted on the surface of Asc l 5 (red color) and come from aspartic (Asp) and glutamic (Glu) acid highlighted in red in the sequence of Asc l 5 showed in Figure S3A, where they represent 19.7% of total amino acids content in the mature sequence. Carboxyl groups of Asp and Glu are able to coordinate to Ca^2+^ [17]. The major coordinating ligands for magnesium as other alkaline-earth metals are carboxyl groups from Asp/Glu, additionally phosphate groups from the nucleic acid backbone and imidazole ring from histidine side chains [18]. In the Asc l 5 model, three binding sites were predicted for Ca^2+^ and one for Mg^2+^, involving residues such as Asp, Glu, His and Lys (Figure S3B). Some of the Asp/Glu predicted in Asc l 5 (Figure S3A) as metal binding residues are conserved in aligned sequences in Figure 1, especially Asp from SXP motif 2 (88D) (Figure S3A). Interestingly, this residue was predicted to be involved in Mg^2+^ binding. Another annotation about the predicted Mg^2+^ binding site is that it is close to one of the Ca^2+^ binding sites where the Glu residue (70E) interacts with both ions (Figure S3B,C). In the Figure S3C, three Ca^2+^ and one Mg^2+^ ions have been added to the Asc l 5 model (the model has been rotated to appreciate residues involved in ion binding). The structure was energy minimized in the presence of all four ions in chimera. In the Figure S3C, Ca^2+^ ions are shown in blue, the Mg^2+^ ion is shown in magenta.

### 2.6. rAsc l 5 Binds IgE, Activates Basophils and Induces Histamine Release

**Figure 4:**
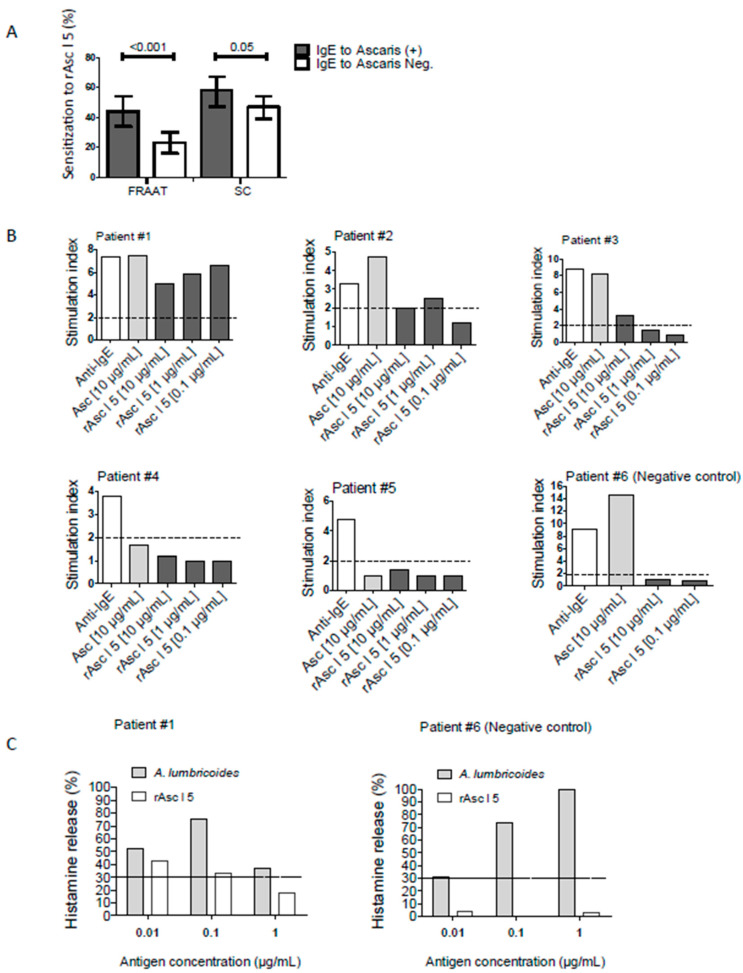
Allergenic properties of rAsc l 5 as evaluated in humans. (**A**) Bar graphs summarizing frequencies of sensitization to rAsc l 5 between individuals sensitized and non-sensitized to *Ascaris*: 254 children from the FRAAT cohort and 298 all-age subjects from Santa Catalina (SC). Error bars indicate 95% confidence intervals. Cut off for IgE sensitization to rAsc l 5 was 0.145 and cut off for IgE sensitization to *Ascaris* was 0.35 kU_A_/L. (**B**) Stimulation index of CD203c based-basophil activation test, including samples stimulated with anti-IgE, *Ascaris lumbricoides* extract (10µg/mL) and rAsc l 5 (10, 1 or 0.1 µg/mL) from five Asc l 5-sensitized patients and one Asc l 5 non-sensitized negative control. (**C**) Percentage of histamine release determined by ELISA in a selected Asc l 5-sensitized patient and the negative control.

We investigated the frequency of IgE sensitization to rAsc l 5 in 254 children from the “Risk Factors for Asthma and Allergy in the Tropics” (FRAAT) cohort and 298 all-age subjects from Santa Catalina (SC). The frequency of IgE-binding to rAsc l 5 in the FRAAT cohort and SC were 31.9% and 52.0% respectively. Sensitization to rAsc l 5 was higher in individuals also sensitized to the *Ascaris* extract (FRAAT: 44% vs. 23%, *p* < 0.001; SC: 58% vs. 47%, *p* = 0.05, Figure 4A). Frequencies of IgE reactivity to rAsc l 5 were also analyzed between asthmatic patients and controls from SC (Table 1), finding no significant difference (58.0% vs. 50.8%, *p* = 0.35).

The immunological characteristics of samples used in CD203 based BAT and histamine release are described in Table 2. Basophil activation results of individual samples are shown in Figure 4B as the stimulation index (SI). rAsc l 5 induced significant upregulation of basophil CD203c expression in 3 out of 5 Asc l 5-sensitized patients (SI range from 2.0 to 6.6). The highest basophil activation value was observed in patient 1 (SI 6.6), like that obtained with *A. lumbricoides* extract (SI 7.5). No activation was observed in the cells from a rAsc l 5 non-sensitized control (patient #6, as negative control). *A. lumbricoides* extract induced basophil activation in all Ascaris-sensitized patients (patients #1, #2 and #3). As expected, anti-human IgE antibodies induced basophil activation that was used as the positive control of the experiment. Histamine release results from a selected patient (patient #1) and the control (patient #6) are shown in Figure 4C. The highest percentage of histamine release in the patient 1 stimulated with rAsc l 5 reached 42.3% while there was no histamine release from the control.

### 2.7. rAsc l 5 Induces Specific IgE Production in Immunized Mice and A Positive Passive Cutaneous Anaphylaxis (PCA) Reaction

**Figure 5:**
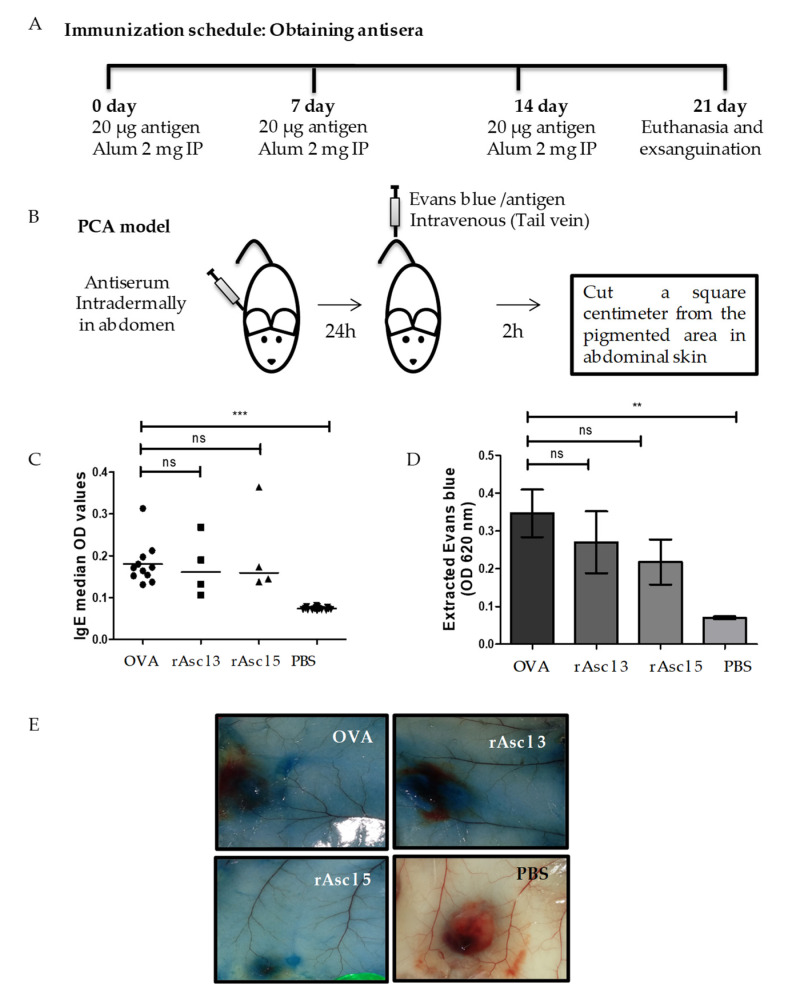
Allergenic properties of rAsc l 5 evaluated in a sensitization and passive cutaneous anaphylaxis (PCA) model (**A**) Immunization schedule of female BALB/c mice using *Ascaris* molecules plus Alum: Asc l 3 and rAsc l 5. Positive control OVA. Negative control PBS. IP: intraperitoneal. (**B**) PCA model in BALB/c mice. (**C**) Strength of the IgE reactivity to OVA, Asc l 3 and rAsc l 5, median values are shown. (**D**) Extracted Evans blue after PCA reaction detected to 620nm showed as mean ± standard error of the mean (SEM) of OD values (OD: Optical density units). Comparison between groups was done by one-way analysis of variance (ANOVA) and Dunnett’s multiple comparison test: ** *p* < 0.05, *** *p* < 0.001, no significance for p > 0.05. ns: not significant). (**E**) Representative pictures after PCA reaction from the pigmented area in abdominal skin.

The immunization protocol to obtain antisera and PCA model is shown in Figure 5A,B. Ova was used as a positive control and induced the strongest IgE responses and extravasated Evans blue levels, followed by *Ascaris* tropomyosin (Asc l 3) and rAsc l 5. However, there were no significant differences among the three responses (Figure 5C,D).

Representative images of PCA reactions show that extravasated Evans Blue is more intense in OVA, Asc l 3 and Asc l 5 groups compared with PBS control group (Figure 5E). These results show that rAsc l 5 can induce specific IgE production and after subsequent rAsc l 5 administration, it induces a type I hypersensitivity reaction.

## 3. Discussion

Research on the relationships between helminthiases and allergic responses is important, not only for getting better knowledge of the pathogenesis of both conditions, but also because in places where they are prevalent, clinical repercussions in the diagnosis and management of asthma and other allergic diseases are expected [19]. Also, allergic reactions could jeopardize the safety of potential helminth vaccines. The analysis of the allergenic composition of *A. lumbricoides* is important for evaluating the role of its allergens on the natural history of asthma and other allergic diseases [9], as well as defining an array of species-specific and cross-reactive molecules for component-resolved diagnosis of allergy [20,21,22]. 

In a previous study we found that *Ascaris* extract had at least 12 IgE-binding components [6], since only three allergens have been described, the complete allergenic composition of this nematode remains to be defined. To identify new allergens, we constructed an *A. lumbricoides* cDNA library and, by using IgE antibodies from *Ascaris*-sensitized subjects, Asc l 5 was discovered. In this study we focused firstly on the physicochemical characterization of Asc l 5, with emphasis on structure and ligand binding characteristics, and secondly, we investigated the frequency of IgE recognition of Asc l 5 and its allergenic activity in humans and in a mouse model of passive cutaneous anaphylaxis.

Asc l 5 is a member of the SXP/RAL-2 protein family, which includes proteins from various nematode species and has amino acid sequence similarity with those of nematode origin only, which suggest that Asc l 5 could be useful for serological diagnosis purposes. However, additional studies are needed to define this aspect. In SXP/RAL-2 protein family some member have being studied as potential vaccine candidates [23,24,25] and others are markers of infection [26,27]. Interestingly, there are 3 allergens of *Anisakis simplex* that belong to this family [28,29,30]. DUF148 domain (Pfam code PF02520) is present in these proteins and comprises two conserved motifs: SXP1 and SXP2 [26], which are present in Asc l 5 (Figure 1). The function of this domain is unknown, but key information comes from Ani s 5, the only member of the SXP/RAL-2 family with 3D structure resolved by NMR composed of six alpha helices with a Calmodulin like fold, with functional studies indicating that Ani s 5 binds with magnesium [14]. Sequence analysis of Asc l 5 and its close homologue Ani s 5 indicated that common structural characteristics might be shared. Asc l 5 has one predicted binding site for magnesium and two for calcium, and our experimental results have shown that structural changes in Asc l 5 are induced by these two metal ions.

Studying the physicochemical properties of allergens helps to understand their biological function and allergenic activities. In addition, it gives the possibility to molecular modifications for experimental or clinical uses. Ani s 5, Ani s 8 and Ani s 9 from *Anisakis simplex* and now Asc l 5 from *A. lumbricoides* are the only allergens that have been identified in the SXP/RAL-2 protein family. The fold of Ani s 5 is remindful of Calmodulin structure which is the primary calcium binding protein in eukaryotic cells. Calmodulin binds Ca^2+^ through a helix-loop-helix structure (EF-hand motif), with highly hydrophobic helices and negative residues [31]. Calmodulin function and structure are influenced by calcium concentration. Folding of its individual domains change significantly in response to Ca^2+^ [15]. Without Ca^2+^, Calmodulin has a “closed” configuration and after Ca^2+^ binding it changes to a dumbbell-shaped “open” configuration, in this motion process becomes exposed to non-polar residues such as methionine that interact with Calmodulin target proteins. These targets are hundreds of intracellular proteins involved in a wide array of functions [32,33].

As described for Ani s 5, our results show that Asc l 5 is a monomeric protein. Some proteins require calcium or magnesium ions to be functional. The binding site is a region in the protein where metal ions will bind as a result of electrostatic interactions with residues of opposite charge through coordination bonds [17]. The 3D structure model of Asc l 5 has negatively charged patches rich in Glu and Asp that provide opposite charge to divalent cations such as typical alkaline-earth metal, Ca^2+^ and Mg^2+^. Highly negative proteins are usually involved in binding to Ca^2+^ and Mg^2+^ [17]. Calcium ions can stabilize the secondary and tertiary structures of proteins that, for example allow binding of other ions [34]. Calcium coordination bond distance is around 2.4–2.5 Å, while for magnesium it is typically 2.08 Å. This agrees with our in in silico analysis where we used a bond distance within the range of 3.5 Å. Our bioinformatics analysis support CD and ANS-experimental results that demonstrated structural changes in rAsc l 5 after adding Ca^2+^ or Mg^2+^, which could be a consequence of its metal binding behavior whereby both ions may act as regulators that reposition/modify folding of secondary structural elements in Asc l 5.

The implications of these observations will serve as a base for future studies and to further analyze and get experimental structural data on the atomic level by X-ray crystallography or NMR spectroscopy and confirm residues involved in metal binding and its stoichiometry, as well as, to study possible effects of one metal ligand on the binding of the other. In addition, since protein ligands can influence allergenicity by affecting allergen tertiary structure and biochemical attributes [35,36,37] the study of structural modifications induced by ligands might help to analyze possible relationships between allergen structure and allergenicity.

rAsc l 5 binds IgE with high frequency in *Ascaris*-sensitized individuals as evaluated in different populations: 44% in FRAAT and 58% in SC. In the SC population, it was possible to evaluate the clinical impact of this sensitization, showing that there is no difference in the degree or frequency of sensitization between asthmatic and non-asthmatic individuals (Table 1). Although the number of cases is small and this is not a main objective of this work, our data suggest that contrary to *Ascaris* tropomyosin, Asc l 5 does not have a significant impact on asthma evolution. However, more studies should be undertaken to address this point, not only in asthma [20] but also to explore the effect of Asc l 5 sensitization on other allergic diseases such as atopic dermatitis, an aspect of ascariasis that have been little investigated [38]. The strength of the IgE response to rAsc l 5 is similar to that observed in other studies against *Ascaris* tropomyosin or ABA-1 [9,20,39], and greater than that detected for glutathione transferase allergen (Asc l 13) [8,20]. In both in vitro and in vivo tests for allergenic activity, rAsc l 5 induces positive basophil activation and histamine release in humans. Also, rAsc l 5 induces specific IgE production and positive PCA reaction with similar strength as that of tropomyosin. All these results from humans and mice suggest that specific IgE to rAsc l 5 can induce allergic inflammation.

ELISA inhibition experiments showed that rAsc l 5 inhibits around 30% of IgE binding capacity of the patient sera directed against *A. lumbricoides* extract, indicating that the response to Asc l 5 may be a third part of the total extract reactivity, supporting the potential usefulness of Asc l 5 in arrays for component resolved diagnosis of *Ascaris* allergy.

In the SXP/RAL-2 protein family there are some antigens that have been proposed as potential vaccine candidates for *Ascaris*-infection prevention with results supported in mouse and pig experimental models, such as As14 [23] and As16 [24,40,41]. Our findings about the allergenic activity of Asc l 5 might have implications for the use of *Ascaris* molecules as potential vaccines against ascariasis and in a general way in the use of vaccines to prevent helminthiases. Considering the allergenic activity of Asc l 5, it is reasonable to think that it could be useful to evaluate the allergenic properties of nematode-candidate antigens before designing vaccines from the SXP/RAL-2 family. To our knowledge, safety of nematode vaccines has been studied only in one phase I trial of a vaccine candidate from *Necator americanus* in which the vaccination of healthy adults living in a hookworm-endemic area of Brazil with a single dose of Na-ASP-2 antigen (Ancylostoma-secreted protein 2) resulted in generalized urticarial reactions in several volunteers [42]. In this study, the authors suggest that in endemic areas nematode infections might induce IgE responses to candidate vaccine antigens and hypersensitivity reactions, highlighting the importance of performing serological studies before the administration of vaccine candidates for preventing helminth infections [42].

In summary, we described the identification and physicochemical characterization of Asc l 5 a new allergen from *A. lumbricoides*. This work contributes to the knowledge of physicochemical properties of SXP/RAL-2 family members and to the growing list of *Ascaris lumbricoides* allergens. Asc l 5 might be useful for diagnosing of *A. lumbricoides* infection and for component-resolved diagnosis of allergy. Asc l 5 is the second member of SXP/RAL-2 protein family that has been physicochemically characterized. The study of biological functions of Asc l 5 and other members of the SXP/RAL-2 protein family may be useful to reveal the role of these molecules in nematodes’ metabolism and host-parasite relationships. Analyzing the possible links between infection and host responses (such as IgE and Th2 immunity) may contribute to understanding the relationship between helminth infections and allergy.

## 4. Materials and Methods

### 4.1. Subjects

The participants were from two well-characterized populations [39,43], here summarized as follows: (1) A population from Santa Catalina (SC, n = 298, aged 1 to 88 years), a small tropical farming/fishing town located at the North of Colombia [39] where current ascariasis was determined by stool analysis with a prevalence of infection of 62.5% [39]. Individuals with available serum samples were included in this study. Using an “International Study of Asthma and Allergies in Childhood” (ISAAC)-based questionnaire previously validated in our country, a ‘case’ of asthma was defined as described previously [44]. Affirmative cases were confirmed by medical evaluation, 50 were asthmatic patients and 248 non-asthmatic controls, recruited from the same neighborhoods [39]; (2) The second population were children of four-year-old (range: 47–57 months) from the FRAAT cohort (n = 254). This is a community-based birth cohort for a prospective follow-up and collection of epidemiological data and biological samples [12,21,43].

### 4.2. Ethical Statement

This study was conducted following the ethical principles for medical research stated in the Declaration of Helsinki. The Bioethics Committee of the University of Cartagena approved the study (Minute 17-05-2012). A full verbal explanation of the investigation was given to each participant and written informed consent was obtained from all subjects or their parents or legal guardians.

### 4.3. IgE Immunoscreening of A. lumbricoides cDNA Library

Total RNA extraction was performed from three *A. lumbricoides* adult females using TRIzol (Gibco, Invitrogen, Carsbald CA, USA). Messenger RNA was purified by a batch method (Oligotex, Qiagen GmbH, Hilden, Germany), and cDNA synthesis was performed using cDNA Synthesis Kit (Agilent) following the manufacturer’s instructions. The cDNA inserts were ligated into the Uni-ZAP XR vector (Agilent) and packaged into bacteriophage Gigapack III Gold (Agilent). Infection of host bacteria was performed in *Escherichia coli* XL1-Blue MRF’. The cDNA library was stored at −80 °C until use [45]. IgE immunoscreening was done using five sera from *A. lumbricoides*-infected children with positive IgE to *Ascaris* (9.81 to 28.6 kU_A_/L), and five negative controls (IgE lower than 0.08 kU_A_/L). Infection of host bacteria with the phages was undertaken at a density of 5000 plaque-forming units per Petri dish (150 mm diameter). Plaques were transferred to nitrocellulose filters (Amersham Corp., Arlington Heights, IL, USA) soaked in isopropyl β-D-1-thiogalactopyranoside (IPTG). The filters were blocked for 1 h with 5% solution of skim milk powder and were washed three times with PBS containing 0.1% Tween 20. They were incubated overnight at room temperature, with the pool of human sera. IgE binding was detected by incubating the membranes with an alkaline phosphatase-conjugated anti-human IgE antibody–(Sigma) diluted 1:500 in PBS containing 0.2% bovine serum albumin (BSA) and 0.05% Tween-20, and developed with 5-bromo-4-chloro-3-indolyl-phosphate/nitro blue tetrazolium (BCIP/NBT) substrate (BIO-RAD) dissolved in alkaline phosphatase buffer (pH 9.5).

### 4.4. Isolation, Sequencing, and Nucleotide Analysis of IgE-Binding Clones

Agar lysis plaques were obtained and placed in 1.5 mL microfuge tubes with 100 µL of SM buffer and stored at 4 °C. For each of the positive clones the screening was repeated to obtain isolated plaques. The DNA inserts were amplified directly by polymerase chain reaction (PCR) with the universal primers “T7 promoter” and “M13 reverse”, selected according to the cloning sites of the phagemid pBluescript SK (-). Conditions for the PCR in a thermocycler S1000 (BIO-RAD) were as follows: initial denaturation of 5 min at 95 °C, followed by 35 cycles of 1 min at 94 °C; 45 s at 52 °C, 1 min at 72 °C and a final extension at 72 °C for 5 min. Analysis of the amplification product was performed in a 1% agarose gel in the presence of SYBR safe DNA gel stain (Invitrogen). Nucleotide sequences were undertaken with the BigDye Terminator v3.1 Cycle Sequencing Kit using capillary electrophoresis and ABI3730xl sequencer. Online bioinformatics tools including Basic Local Alignment Search Tool (BLASTN) and open reading frame (ORF) finder tool (http://www.ncbi.nlm.nih.gov/gorf/gorf.html) were used to identify the nucleotide sequences and determine similarities with previously reported sequences in GenBank. Clustal Omega (https://www.ebi.ac.uk/Tools/msa/clustalo/) was used to multiple alignments of cDNA and protein sequences and SignalP-5.0 Server (http://www.cbs.dtu.dk/services/SignalP/) to analyze the presence of a signal peptide on the protein sequence.

### 4.5. Cloning, Expression, and Purification of Recombinant Asc l 5

The cDNA sequence coding for Asc l 5 protein was subcloned without signal peptide into the expression vector pET-45b+ (GenScript, USA) and *E. coli* Origami (DE3) competent cells were transformed by electroporation. The *E. coli* cultures were grown overnight in Luria–Bertani medium containing 100 mg/L ampicillin at 37 °C. Expression of the recombinant protein was induced by adding IPTG to a final concentration of 1 mM at an OD_600_ of 0.5. After cultivation for additional 5 h at 37 °C, *E. coli* cultures were centrifuged (30 min, 3500 rpm, 4 °C). Induced cultures were re-suspended in native buffer (50 mM NaH_2_PO_4_, 300 mM NaCl), incubated with lysozyme (1 mg/mL, 30 min on ice) and sonicated. Lysates were incubated with Nickel-Nitrilotriacetic Acid (Ni-NTA) resin (Invitrogen) for one hour, washed with native buffer plus 20 mM imidazole, and eluted with native buffer plus 250 mM imidazole as a 6xHis-tagged protein [8]. The purified recombinant was directly subjected to SDS-PAGE analysis.

### 4.6. A. lumbricoides Extract Preparation, Enzyme-Linked Immunosorbent Assay (ELISA) Inhibition and Western Blot (WB)

Adult *A. lumbricoides* worms were washed in sterile saline solution (0.9% p/v of sodium chloride) with penicillin and streptomycin, then washed with PBS and cut in small pieces that were frozen with liquid nitrogen and homogenized using mortar and pestle. After the liquid nitrogen was evaporated completely, macerated worms were diluted in PBS in a 1:10 proportion (1g of worms in 10 mL) and centrifuged. The supernatant (extract) was dialyzed in water using a 3500 Dalton molecular weight cutoff membrane (Spectra/Por^®^). Protein concentration (1.3 mg/mL) was determined by Bradford (Thermo). The lyophilized extract was kept at −20 °C until used. For ELISA inhibition between rAsc l 5 and *A. lumbricoides* extract, a pool of serum samples (1:5 dilution) from *Ascaris*-sensitized patients (Table S1) was incubated with the inhibitors rAsc l 5, *A. lumbricoides* extract or BSA (Bovine serum albumin) respectively at different concentrations (0.001, 0.01, 0.1, 1, 10, 100 and 1000 µg/mL). After adsorption during 10 h at 4 °C, 100 µL of the inhibited pool was loaded into each well coated with *A. lumbricoides* extract on solid phase. IgE-binding was measured by ELISA, and the results were expressed in percentage of inhibition, calculated as follows: percent of inhibition = OD without inhibitor—OD with inhibitor/OD without inhibitor X 100. BSA was the nonrelated inhibitor control [6]. WB using sera from *Ascaris*-sensitized patients was undertaken to evaluate IgE-reactivity of the recombinant Asc l 5. The protocol was performed as described before [9]. A pool of sera from 5 *Ascaris*-sensitized patients with specific IgE to *Ascaris* spp. ranged from 3.88 to 8.54 kU_A_/L as determined by ImmunoCAP and 5 negative controls with specific IgE to *Ascaris* spp. bellow 0.08 kU_A_/L and PBS buffer control were used to evaluate reactivity of rAsc l 5. WB using sera from mice immunized with rAsc l 5 (see below) was used to identify its native counterpart (nAsc l 5) in the *A. lumbricoides* extract. For gel electrophoresis, 10 µg of *A. lumbricoides* extract and 0.375 µg of rAsc l 5 were applied to each well of 12% SDS-PAGE. After SDS-PAGE, proteins were blotted onto a nitrocellulose membrane (Whatman, Maidstone, UK) and incubated with sera pool of 4 rAsc l 5-immunized mice diluted 1:200 and bound IgG1 was detected using a rat AP-conjugated anti-mouse IgG1 antibody (1:2000, Southern Biotech) [46].

### 4.7. Peptide Analysis by Nano-Liquid Chromatography–Tandem Mass Spectrometry (LC-MS/MS)

5 μL of *A. lumbricoides* extract (1000 μg/mL) were digested with the ProteoExtract All-in-One Trypsin Digestion Kit (EMD Millipore, Billerica, MA, USA) and desalted using C18ZipTips (EMD Millipore, Billerica, MA, USA). Resulting peptides were separated by reverse-phase nano-high performance liquid chromatography (HPLC, Dionex Ultimate 3000, Thermo Fisher Scientific, Bremen, Germany, column: PepSwift Monolithic Nano Column, 100 μM × 25 cm, Dionex). The column was developed with an acetonitrile gradient (Solvent A: 0.1% (*v*/*v*) FA/0.01% (*v*/*v*) TFA/5% (*v*/*v*) ACN; solvent B: 0.1% (*v*/*v*) FA/0.01% (*v*/*v*) TFA/90% (*v*/*v*) ACN; 5–45% B in 60 min) at a flow rate of 1 μL/min at 55 °C). The HPLC was directly coupled via nano electrospray to a Q Exactive Orbitrap mass spectrometer (Thermo Fisher Scientific). Capillary voltage was 2 kV. For peptide assignment, a top 12 MS/MS method was used with the normalized fragmentation energy set to 27%. Proteins were identified with PEAKS Studio 8 (Bioinformatics Solutions, Waterloo, Canada), using the UniProt (SwissProt/TrEMBL) sequence database. For the identification of post-translational modifications and amino acid exchanges, the PTM and Spider modules of PEAKS Studio were used. Only peptides with high confidence scores (−10lg*p* ≥ 35, corresponding to false discovery rate (FDR) < 0.5%) were considered in the database searches.

### 4.8. Size Exclusion Chromatography, Amino Acid Composition, Aggregation Behavior and Fourier Transform Infrared (FTIR) Spectrosocpy Experiments

An additional purification step of rAsc l 5 was performed by size exclusion chromatography in 5 mM sodium phosphate buffer pH 6.8 using a Superdex 75 10/300 GL column (GE Healthcare Life Sciences) using an ÄKTA pure chromatography system (GE Healthcare Life Sciences). Amino acid composition and protein concentration were determined by amino acid analyses according to the PicoTagTM method (Waters, Milford, MA, USA) using a (HP) 1100 HPLC system (Hewlett-Packard, San Jose, CA, USA) equipped with a 3.9 × 150 mm Nova-pak C18 column (Waters). The aggregation behavior was evaluated by dynamic light scattering (DLS) using a DLS 802 system (Viscotek Corp., Houston, TX, US). Data of 10 independently recorded measurements were evaluated using the OmniSize™ software (Viscotek Corp.) and displayed as mass weighted distribution of the hydrodynamic radius. For FTIR experiments, the spectra of rAsc l 5 (1000 μg/mL) were recorded at a constant temperature (25 °C) using an AquaSpec transmission cell adapted to a Tensor II Confocheck FTIR system (Bruker Optics Inc., Billerica, MA, USA). The second derivative was calculated of the amide 1 band and the curve was smoothed by applying the Savitzky–Golay algorithm with 25 smoothing points. Spectra were vector-normalized, and baseline corrected (background signal of the buffer solution was subtracted). For the analysis of secondary structural elements, a Quant2 method provided by Confocheck (Bruker Optics Inc.) was used.

### 4.9. Calcium and Magnesium-Binding Experiments and Circular Dichroism (CD) Spectroscopy

ANS fluorescence experiments were performed in duplicates and recorded using an Infinite^®^ 200 PRO Series plate reader (Tecan Trading AG, Switzerland) at room temperature and excitation wavelength of 370 nm. rAsc l 5 was prepared in 10 mM Tris buffer at final concentration of 4 μM. CaCl_2_ or MgCl_2_ were prepared in serial two-fold dilutions starting from 400 mM and incubated with rAsc l 5 for 1 h. ANS was added at final concentration of 50 μM. Fluorescence emission spectra were obtained between 400 and 600 nm and represented as area under the curve. CD experiments were recorded to evaluate changes in secondary structure elements of rAsc l 5 in presence of CaCl_2_ or MgCl_2_ at 20 °C. CD spectra were recorded using a JASCO J-815 spectropolarimeter fitted with a PTC-423S Peltier type single position cell holder (Jasco, Tokyo, Japan), in 10 mM K_2_HPO_4_/KH_2_PO_4_. Samples were measured from 190–260 nm at resolution of 1 nm with 1 nm bandwidth and a scanning speed of 1 nm/s. Five spectra were averaged and background of the buffer solution was subtracted. Data were presented as mean residue molar ellipticity. Optimal ratios for CD experiments were selected from previous ANS assays where significant changes in fluorescence intensity were detected (4 µM rAsc l 5/50 mM CaCl_2_ or 4 µM rAsc l 5/150 mM MgCl_2_). Final concentrations for CD experiments were: 100 µg/mL of rAsc l 5 (corresponding to was 5.9 μM), 74 mM CaCl_2_ or 221 mM MgCl_2_, as needed.

### 4.10. Modeling of Three-Dimensional Structure of Asc l 5 and Ca^2+^ and Mg^2+^ Binding Sites

A 3D-homology model of Asc l 5 was based on the known NMR structure of Ani s 5. Asc l 5 models were calculated with MODELLER [47] using all NMR-models from PDB entry 2MAR as templates. The best model was selected with the model scoring option of MAESTRO [48]. The figures of Asc l 5 structure (ribbon and electrostatic surface representations) were done by UCSF Chimera [49]. Electrostatics was calculated with APBS [50]. For prediction of Ca^2+^ and Mg^2+^ binding sites, metal ion-binding (MIB) site prediction and docking server (http://bioinfo.cmu.edu.tw/MIB/) was used, which predicts metal binding sites using protein three-dimensional structure. MIB server was based on the local structural comparison between metal ions-binding residue templates that include residues coordinated to metal ions within 3.5 Å and the 3D-homology model of Asc l 5 [51] by using the fragment transformation method [52].

### 4.11. Specific IgE

Specific IgE antibody levels to *Ascaris* spp, *D. pteronyssinus* and *B. tropicalis* extracts were measured using ImmunoCAP system (Thermo Fisher Scientific), following the technical instructions of the manufacturer. Specific IgE levels equal to or greater than 0.35 kU_A_/L were considered positive. For indirect ELISA serum samples, conjugate dilutions and antigen concentrations were obtained by titration. Each well was coated with 1 µg of antigen diluted in sodium carbonate/bicarbonate buffer. ELISA was performed as described before [20]. Cut-off value to define positive or negative IgE responses to rAsc l 5 was calculated as the mean optical density (OD) of 9 negative controls + 3 SD. The cut-off value for IgE anti-rAsc l 5 was 0.145. Sensitization was defined as a positive specific IgE result detected by ELISA or ImmunoCAP to any extract or to the recombinant allergen.

### 4.12. Basophil Activation Test

The basophil activation test was performed as recommended by the manufacturer of the assay Allergenicity Kit (Beckman Coulter, Inc. CA, USA) and measured by flow cytometry. Peripheral blood samples were collected from five rAsc l 5-sensitized individuals and one rAsc l 5-non sensitized control in endotoxin-free EDTA tubes. Aliquots (50 µL) of the blood were incubated with 10 µl of PBS (unstimulated control), 10 µL of allergenicity positive control (Anti-IgE) or 10 µL of serial dilutions of allergens (to final concentration of 0.1 µg/mL, 1 µg/mL and 10 µg/mL) for 15 min at 37 °C. Basophils were gated based on the expression of CRTh2 marker and activation was assessed by detection of the activation marker CD203c using phycoerythrin-conjugated CD203c mAb 97A6 in a FACSAria™ III cytometer (BD Biosciences). Cytometry data analysis was undertaken using FlowJo V10 (Tree Star, Inc., Ashland, OR, USA). In each assay at least 500 basophils were counted. Allergen-induced upregulation of CD203c was expressed as the stimulation index (SI), calculated using mean fluorescence intensities (MFI) obtained with stimulated sample (MFI stim) divided by unstimulated sample (MFI control), SI = MFI stim/MFI control. SI ≥ 2.0 was considered positive [53,54].

### 4.13. Histamine Release

Heparinized blood was drawn from samples using BD Vacutainers (Becton Dickinson, Franklin Lakes, NJ, USA). Basophil histamine release was induced by incubating 200 µL of fresh blood with 200 µL of serial concentrations (to final concentration of 0.01, 0.1, 1 µg/mL) of rAsc l 5 or *A. lumbricoides* extract. Allergen dilution buffer and anti-human IgE positive control were used as described by the manufacturer (Histamine Release kit; IBL International GmbH, Hamburg, Germany). Histamine concentrations were quantified by ELISA (IBL International GmbH, Hamburg, Germany). Total histamine release was determined in cells treated with hypotonic solution and regarded as 100% release. Spontaneous release was calculated in cells treated with allergen dilution buffer, and the result was subtracted from cells treated with allergens. Absorbance was measured at 450 nm. Percent of allergen-induced release was calculated in relation to the total histamine release [9]. 

### 4.14. Production of Antisera against rAsc l 5, Antibody Determinations and PCA Model

Polyclonal antisera to rAsc l 5 were raised in 6 to 8-week-old female BALB/c mice (Instituto Nacional de Salud, INS, Bogotá, Colombia). Mice received intraperitoneal injections of rAsc l 5 (20 µg) suspension with aluminum hydroxide 2 mg (Thermo Fisher Scientific) three times in a seven-day interval [55] and blood sampled 7 days after the last injection. Mice were also immunized with the HDM cross-reactive allergen tropomyosin (Asc l 3) [9]. Solutions were prepared in PBS (Dulbecco’s Phosphate-Buffered Saline, Thermo Fisher Scientific) before use and mixed thoroughly to ensure homogeneity. OVA-sensitized animals were the positive controls for these experiments [56] and PBS-treated mice the negative controls. Mice were kept under specific pathogen-free environment (22 °C, 50–60% humidity, and 12-h light/dark cycle) and fed with standard pellet diet and drinking water ad libitum [45]. Animal experiments were performed in accordance with institutional protocols (University of Cartagena, Minutes 117, 13-03-2019). Antibody responses of animals were evaluated by ELISA. MaxisorpTM microtiter plates were coated with the antigens (0.5 µg/well) by overnight incubation at 4 °C and washed 4 times with PBS 0.1% Tween 20. Wells were then blocked with PBS 1% BSA for 3 h at room temperature. Plates were washed (5X) and incubated overnight with diluted plasma samples (1:10) at 4 °C. After 5 washes, wells were incubated with biotin labeled anti-mouse IgE 1:1000 (Clone 2363, eBioscience), 1 h at room temperature (RT). After 5 washes, streptavidin-alkaline-phosphatase 1:4000 (Sigma-Aldrich, Saint Louis, MO, USA) was added and incubated an additional hour. *p*-nitrophenyl phosphate (1 mg/mL) was used as substrate solution. Optical densities were read at 405 nm in a spectrophotometer [55]. To increase the sensitivity of the IgE ELISA, IgG was depleted from serum by incubation with protein G sepharose [57]. The PCA model was performed as follows. Naive 6 to 8-week-old female BALB/c mice were injected intradermally with 100 µL of plasma from immunized mice into abdominal skin. Twenty-four hours later, each mouse was injected via the tail vein with 200 µL containing 25 µg of *Ascaris* molecules or PBS control, plus 0.5% Evan’s Blue (PanReac AppliChem) dissolved in PBS. After 2 h, the mice were euthanized. One square centimeter was cut from the pigmented area in each mouse. The removed skin was incubated with formamide, and then the extravasated Evans blue dye was extracted. The amount of dye absorbance at 620 nm was measured in a spectrophotometer (Spectra Max 250; Molecular Devices, Sunnyvale, CA, USA) [58].

### 4.15. Statistics

Analyses were conducted using SPSS version 20.0 (SPSS, Chicago, IL, USA) and GRAPHPAD PRISM version 5.01 for Windows (GraphPad Software, San Diego, CA, USA). IgE values of individual participants were not normally distributed; therefore, they were reported as median values and interquartile ranges (IC). Differences between proportions of subjects sensitized were analyzed by a Pearson chi-square test. The Mann–Whitney U-test was used for comparison of continuous variables as needed. For all analyses, *p* values < 0.05 (two tailed) were considered significant. For comparison of means of more than two groups, one-way ANOVA and Dunnett’s multiple comparison test were used. In this case mean ± standard error of the mean (SEM) was used for each group.

## Figures and Tables

**Table 1:** Serological and parasitological variables, Santa Catalina (SC).

Variables	Asthmatics, *n* (%)	Non Asthmatics, *n* (%)	*p* Value
	***n = 50***	***n = 248***	
Asc l 5 sensitization ^1^	29 (58.0%)	126 (50.8%)	0.353
IgE to Asc l 5, OD ^2^	0.154 (0.114–0.253)	0.146 (0.115–0.233)	0.388
*Ascaris* spp. sensitization ^1^	26 (50%)	104 (42%)	0.190
IgE to *Ascaris* spp ^2^	0.444 (0.036–1.475)	0.251 (0.07–1.134)	0.821
	***n = 48***	***n = 242***	
*A. lumbricoides* by stool examination ^1^	27 (56.3%)	168 (69.4%)	0.076
*A. lumbricoides* epg ^2^	546 (0–2047)	689 (0-2340)	0.197
	***n = 49***	***n = 236***	
Positive SPT to *Ascaris* ^1^	13 (26.5%)	30 (12.7%)	0.014

^1^ Determined as positive or negative n (%). *p* values by Pearson chi-square test. Cut off for rAsc l 5 sensitization was 0.145. Cut off for *Ascaris* spp. sensitization was 0.35 kU/l. Skin prick test (SPT) was considered positive if the mean diameter of the wheal at 15 min was > 3 mm than the negative control. ^2^ Medians (interquartile range, 25th–75th). *p* values were calculated using the Mann–Whitney U-test. OD: Optical density units. epg: Eggs per gram of faeces.

**Table 2:** Immunological characteristics of samples used in basophil activation test and histamine release.

Patients						
*Data*	#1	#2	#3	#4	#5	#6
Code	ST371	ST581	ASC217	ST383	ST438	ASC040 ^ɫ^
Diagnosis	Asthma	Parasited	Asthma	Rhinitis	Asthma	Asthma
IgE to rAsc l 5 ^1^	0.29	0.51	0.29	0.38	1.20	0.12
IgE to *Ascaris* ^2^	16.2	8.19	3.64	0.30	0.03	9.02
IgE to *Blomia* ^2^	22.80	0.67	0.48	2.01	0.10	1.33
IgE to Dp. ^2^	2.20	0.10	0.31	7.04	0.02	1.16
Total IgE ^2^	642.6	727.1	588.3	478.7	228.5	975.8
Gender	F	F	M	F	F	F
Age	50	61	35	36	3	28
*Stimulation index (SI) of CD203 based-basophil activation test*						
Anti-IgE	7.4	3.3	8.7	3.8	4.8	9.0
rAsc l 5 10 µg/mL	5.0	2.0	3.2	1.2	1.4	1.0
rAsc l 5 1 µg/mL	5.9	2.5	1.4	1.0	1.0	nd
rAsc l 5 0.1 µg/mL	6.6	1.2	0.9	1.0	1.0	0.9
*Ascaris* 10 µg/mL	7.5	4.7	8.1	1.7	1.0	14.6
*Percentage of histamine release (%)*						
*Ascaris* 0.01 µg/mL	52.2	nd	nd	nd	nd	31.0
*Ascaris* 0.1 µg/mL	75.2	nd	nd	nd	nd	73.0
*Ascaris* 1 µg/mL	37.0	nd	nd	nd	nd	100
rAsc l 5 0.01 µg/mL	42.3	nd	nd	nd	nd	4
rAsc l 5 0.1 µg/mL	33.2	nd	nd	nd	nd	0.4
rAsc l 5 1 µg/mL	18.0	nd	nd	nd	nd	3

^1^ OD: Optical density units. As determined by ELISA. ^2^ kU/l as determined by ImmunoCAP. Dp: *Dermatophagoides pteronyssinus*. ^ɫ^ rAsc l 5 non-sensitized control. nd. Not done.

## Supplementary Materials

The following are available online at https://www.mdpi.com/1422-0067/21/24/9761/s1.

## Abbreviations

IPTG	isopropyl β-D-1-thiogalactopyranoside
PBS	phosphate buffered saline
SDS-PAGE	sodium dodecyl sulfate polyacrylamide gel electrophoresis
nano-LC-MS/MS	Nanoscale liquid chromatography coupled to tandem mass spectrometry
CD	Circular dichroism
DLS	Dynamic light scattering
ELISA	Enzyme-linked immunosorbent assay
SPT	skin prick test
E. coli	Escherichia coli
LB	Luria Bertani
DUF	domain of unknown function
HDM	House dust mite
IUIS	International Union of Immunological Societies

## References

[1] Hagel I., Cabrera M., Hurtado M.A., Sanchez P., Puccio F., Di Prisco M.C., Palenque M. (2007). Infection by Ascaris lumbricoides and bronchial hyper reactivity: An outstanding association in Venezuelan school children from endemic areas. Acta Trop..

[2] Moncayo A.L., Vaca M., Oviedo G., Workman L.J., Chico M.E., Platts-Mills T.A., Rodrigues L.C., Barreto M.L., Cooper P.J. (2013). Effects of geohelminth infection and age on the associations between allergen-specific IgE, skin test reactivity and wheeze: A case-control study. Clin. Exp. Allergy J. Br. Soc. Allergy Clin. Immunol..

[3] Caraballo L. (2018). The tropics, helminth infections and hygiene hypotheses. Expert Rev. Clin. Immunol..

[4] Weatherhead J.E., Porter P., Coffey A., Haydel D., Versteeg L., Zhan B., Gazzinelli Guimaraes A.C., Fujiwara R., Jaramillo A.M., Bottazzi M.E. (2018). Ascaris Larval Infection and Lung Invasion Directly Induce Severe Allergic Airway Disease in Mice. Infect. Immun..

[5] Acevedo N., Caraballo L. (2011). IgE cross-reactivity between Ascaris lumbricoides and mite allergens: Possible influences on allergic sensitization and asthma. Parasite Immunol..

[6] Acevedo N., Sanchez J., Erler A., Mercado D., Briza P., Kennedy M., Fernandez A., Gutierrez M., Chua K.Y., Cheong N. (2009). IgE cross-reactivity between Ascaris and domestic mite allergens: The role of tropomyosin and the nematode polyprotein ABA-1. Allergy.

[7] Caraballo L., Acevedo N., Zakzuk J. (2019). Ascariasis as a model to study the helminth/allergy relationships. Parasite Immunol..

[8] Acevedo N., Mohr J., Zakzuk J., Samonig M., Briza P., Erler A., Pomes A., Huber C.G., Ferreira F., Caraballo L. (2013). Proteomic and immunochemical characterization of glutathione transferase as a new allergen of the nematode Ascaris lumbricoides. PLoS ONE.

[9] Acevedo N., Erler A., Briza P., Puccio F., Ferreira F., Caraballo L. (2011). Allergenicity of Ascaris lumbricoides tropomyosin and IgE sensitization among asthmatic patients in a tropical environment. Int. Arch. Allergy Immunol..

[10] Caraballo L., Coronado S. (2018). Parasite allergens. Mol. Immunol..

[11] Caraballo L., Acevedo N. (2011). New Allergens of Relevance in Tropical Regions: The Impact of Ascaris lumbricoides Infections. World Allergy Organ. J..

[12] Zakzuk J., Acevedo N., Cifuentes L., Bornacelly A., Sanchez J., Ahumada V., Ring J., Ollert M., Caraballo L. (2013). Early life IgE responses in children living in the tropics: A prospective analysis. Pediatr. Allergy Immunol. Off. Publ. Eur. Soc. Pediatric Allergy Immunol..

[13] Caraballo L. (2017). Mite allergens. Expert Rev. Clin. Immunol..

[14] Garcia-Mayoral M.F., Trevino M.A., Perez-Pinar T., Caballero M.L., Knaute T., Umpierrez A., Bruix M., Rodriguez-Perez R. (2014). Relationships between IgE/IgG4 epitopes, structure and function in Anisakis simplex Ani s 5, a member of the SXP/RAL-2 protein family. PLoS Negl. Trop. Dis..

[15] Stigler J., Rief M. (2012). Calcium-dependent folding of single calmodulin molecules. Proc. Natl. Acad. Sci. USA.

[16] Gabellieri E., Strambini G.B. (2006). ANS fluorescence detects widespread perturbations of protein tertiary structure in ice. Biophys. J..

[17] Kirberger M., Yang J.J., Kretsinger R.H., Uversky V.N., Permyakov E.A. (2013). Calcium-Binding Protein Site Types. Encyclopedia of Metalloproteins.

[18] Zheng H., Cooper D.R., Porebski P.J., Shabalin I.G., Handing K.B., Minor W. (2017). CheckMyMetal: A macromolecular metal-binding validation tool. Acta Crystallogr. Sect. D Struct. Biol..

[19] Caraballo L., Zakzuk J., Lee B.W., Acevedo N., Soh J.Y., Sanchez-Borges M., Hossny E., Garcia E., Rosario N., Ansotegui I. (2016). Particularities of allergy in the Tropics. World Allergy Organ. J..

[20] Ahumada V., Garcia E., Dennis R., Rojas M.X., Rondon M.A., Perez A., Penaranda A., Barragan A.M., Jimenez S., Kennedy M.W. (2015). IgE responses to Ascaris and mite tropomyosins are risk factors for asthma. Clin. Exp. Allergy J. Br. Soc. Allergy Clin. Immunol..

[21] Zakzuk J., Mercado D., Bornacelly A., Sanchez J., Ahumada V., Acevedo N., Caraballo L. (2019). Hygienic conditions influence sensitization to Blomia tropicalis allergenic components: Results from the FRAAT birth cohort. Pediatr. Allergy Immunol. Off. Publ. Eur. Soc. Pediatr. Allergy Immunol..

[22] Buendia E., Zakzuk J., Mercado D., Alvarez A., Caraballo L. (2015). The IgE response to Ascaris molecular components is associated with clinical indicators of asthma severity. World Allergy Organ. J..

[23] Tsuji N., Suzuki K., Kasuga-Aoki H., Matsumoto Y., Arakawa T., Ishiwata K., Isobe T. (2001). Intranasal immunization with recombinant Ascaris suum 14-kilodalton antigen coupled with cholera toxin B subunit induces protective immunity to A. suum infection in mice. Infect. Immun..

[24] Wei J., Versteeg L., Liu Z., Keegan B., Gazzinelli-Guimaraes A.C., Fujiwara R.T., Briggs N., Jones K.M., Strych U., Beaumier C.M. (2017). Yeast-expressed recombinant As16 protects mice against Ascaris suum infection through induction of a Th2-skewed immune response. PLoS Negl. Trop. Dis..

[25] Fujiwara R.T., Zhan B., Mendez S., Loukas A., Bueno L.L., Wang Y., Plieskatt J., Oksov Y., Lustigman S., Bottazzi M.E. (2007). Reduction of worm fecundity and canine host blood loss mediates protection against hookworm infection elicited by vaccination with recombinant Ac-16. Clin. Vaccine Immunol..

[26] Rao K.V., Eswaran M., Ravi V., Gnanasekhar B., Narayanan R.B., Kaliraj P., Jayaraman K., Marson A., Raghavan N., Scott A.L. (2000). The Wuchereria bancrofti orthologue of Brugia malayi SXP1 and the diagnosis of bancroftian filariasis. Mol. Biochem. Parasitol..

[27] Klion A.D., Vijaykumar A., Oei T., Martin B., Nutman T.B. (2003). Serum immunoglobulin G4 antibodies to the recombinant antigen, Ll-SXP-1, are highly specific for Loa loa infection. J. Infect. Dis..

[28] Kobayashi Y., Ishizaki S., Shimakura K., Nagashima Y., Shiomi K. (2007). Molecular cloning and expression of two new allergens from Anisakis simplex. Parasitol. Res..

[29] Rodriguez-Perez R., Moneo I., Rodriguez-Mahillo A., Caballero M.L. (2008). Cloning and expression of Ani s 9, a new Anisakis simplex allergen. Mol. Biochem. Parasitol..

[30] Kobayashi Y., Shimakura K., Ishizaki S., Nagashima Y., Shiomi K. (2007). Purification and cDNA cloning of a new heat-stable allergen from Anisakis simplex. Mol. Biochem. Parasitol..

[31] Finn B.E., Forsen S. (1995). The evolving model of calmodulin structure, function and activation. Structure.

[32] Yamniuk A.P., Vogel H.J. (2004). Calmodulin’s flexibility allows for promiscuity in its interactions with target proteins and peptides. Mol. Biotechnol..

[33] Yap K.L., Kim J., Truong K., Sherman M., Yuan T., Ikura M. (2000). Calmodulin target database. J. Struct. Funct. Genom..

[34] Schneider L.K., Einsle O. (2016). Role of Calcium in Secondary Structure Stabilization during Maturation of Nitrous Oxide Reductase. Biochemistry.

[35] Dubiela P., Aina R., Polak D., Geiselhart S., Humeniuk P., Bohle B., Alessandri S., Del Conte R., Cantini F., Borowski T. (2017). Enhanced Pru p 3 IgE-binding activity by selective free fatty acid-interaction. J. Allergy Clin. Immunol..

[36] Dubiela P., Del Conte R., Cantini F., Borowski T., Aina R., Radauer C., Bublin M., Hoffmann-Sommergruber K., Alessandri S. (2019). Impact of lipid binding on the tertiary structure and allergenic potential of Jug r 3, the non-specific lipid transfer protein from walnut. Sci. Rep..

[37] Engel E., Richter K., Obermeyer G., Briza P., Kungl A.J., Simon B., Auer M., Ebner C., Rheinberger H.J., Breitenbach M. (1997). Immunological and biological properties of Bet v 4, a novel birch pollen allergen with two EF-hand calcium-binding domains. J. Biol. Chem..

[38] Silva M.T., Costa V.A., Pereira T.G., Sales I.R., Silva S.F., Maciel M.A., Malagueño E., Souza V.M. (2012). Severity of atopic dermatitis and Ascaris lumbricoides infection: An evaluation of CCR4+ and CXCR3+ helper T cell frequency. Rev. Soc. Bras. Med. Trop..

[39] Zakzuk J., Casadiego S., Mercado A., Alvis-Guzman N., Caraballo L. (2018). Ascaris lumbricoides infection induces both, reduction and increase of asthma symptoms in a rural community. Acta Trop..

[40] Matsumoto Y., Suzuki S., Nozoye T., Yamakawa T., Takashima Y., Arakawa T., Tsuji N., Takaiwa F., Hayashi Y. (2009). Oral immunogenicity and protective efficacy in mice of transgenic rice plants producing a vaccine candidate antigen (As16) of Ascaris suum fused with cholera toxin B subunit. Transgenic Res..

[41] Tsuji N., Miyoshi T., Islam M.K., Isobe T., Yoshihara S., Arakawa T., Matsumoto Y., Yokomizo Y. (2004). Recombinant Ascaris 16-Kilodalton protein-induced protection against Ascaris suum larval migration after intranasal vaccination in pigs. J. Infect. Dis..

[42] Diemert D.J., Pinto A.G., Freire J., Jariwala A., Santiago H., Hamilton R.G., Periago M.V., Loukas A., Tribolet L., Mulvenna J. (2012). Generalized urticaria induced by the Na-ASP-2 hookworm vaccine: Implications for the development of vaccines against helminths. J. Allergy Clin. Immunol..

[43] Acevedo N., Sanchez J., Zakzuk J., Bornacelly A., Quiroz C., Alvarez A., Puello M., Mendoza K., Martinez D., Mercado D. (2012). Particular characteristics of allergic symptoms in tropical environments: Follow up to 24 months in the FRAAT birth cohort study. BMC Pulm. Med..

[44] Dennis R.J., Caraballo L., Garcia E., Rojas M.X., Rondon M.A., Perez A., Aristizabal G., Penaranda A., Barragan A.M., Ahumada V. (2012). Prevalence of asthma and other allergic conditions in Colombia 2009-2010: A cross-sectional study. BMC Pulm. Med..

[45] Coronado S., Barrios L., Zakzuk J., Regino R., Ahumada V., Franco L., Ocampo Y., Caraballo L. (2017). A recombinant cystatin from Ascaris lumbricoides attenuates inflammation of DSS-induced colitis. Parasite Immunol..

[46] Wolf M., Twaroch T.E., Huber S., Reithofer M., Steiner M., Aglas L., Hauser M., Aloisi I., Asam C., Hofer H. (2017). Amb a 1 isoforms: Unequal siblings with distinct immunological features. Allergy.

[47] Webb B., Sali A. (2016). Comparative Protein Structure Modeling Using MODELLER. Curr. Protoc. Bioinform..

[48] Laimer J., Hofer H., Fritz M., Wegenkittl S., Lackner P. (2015). MAESTRO--multi agent stability prediction upon point mutations. BMC Bioinform..

[49] Pettersen E.F., Goddard T.D., Huang C.C., Couch G.S., Greenblatt D.M., Meng E.C., Ferrin T.E. (2004). UCSF Chimera--a visualization system for exploratory research and analysis. J. Comput. Chem..

[50] Jurrus E., Engel D., Star K., Monson K., Brandi J., Felberg L.E., Brookes D.H., Wilson L., Chen J., Liles K. (2018). Improvements to the APBS biomolecular solvation software suite. Protein Sci. A Publ. Protein Soc..

[51] Kumar R., Breindel C., Saraswat D., Cullen P.J., Edgerton M. (2017). Candida albicans Sap6 amyloid regions function in cellular aggregation and zinc binding, and contribute to zinc acquisition. Sci. Rep..

[52] Lin Y.F., Cheng C.W., Shih C.S., Hwang J.K., Yu C.S., Lu C.H. (2016). MIB: Metal Ion-Binding Site Prediction and Docking Server. J. Chem. Inf. Modeling.

[53] MacGlashan D.W. (2013). Basophil activation testing. J. Allergy Clin. Immunol..

[54] Martinez D., Munera M., Cantillo J.F., Wortmann J., Zakzuk J., Keller W., Caraballo L., Puerta L. (2019). An Engineered Hybrid Protein from Dermatophagoides pteronyssinus Allergens Shows Hypoallergenicity. Int. J. Mol. Sci..

[55] Zakzuk J., Benedetti I., Fernandez-Caldas E., Caraballo L. (2014). The influence of chitin on the immune response to the house dust mite allergen Blo T 12. Int. Arch. Allergy Immunol..

[56] Chen C., Sun N., Li Y., Jia X. (2013). A BALB/c mouse model for assessing the potential allergenicity of proteins: Comparison of allergen dose, sensitization frequency, timepoint and sex. Food Chem. Toxicol. Int. J. Publ. Br. Ind. Biol. Res. Assoc..

[57] Lehrer S.B., Reish R., Fernandes J., Gaudry P., Dai G., Reese G. (2004). Enhancement of murine IgE antibody detection by IgG removal. J. Immunol. Methods.

[58] Evans H., Killoran K.E., Mitre E. (2014). Measuring local anaphylaxis in mice. J. Vis. Exp..

