# Supplementary material for: Identification and Physicochemical Characterization of a New Allergen from Ascaris lumbricoides
Source: Int J Mol Sci. 2020 Dec 21;21(24):9761. doi: 10.3390/ijms21249761 (PMC7767342; doi:10.3390/ijms21249761)
Supplement: Supplementary file 1 [file ijms-21-09761-s001.pdf]

**Supplementary material**

**Table S1** Description of pool sera used in ELISA inhibition

| Code | Gender | Age | Origin | Diagnosis | IgE to rAsc l 5 [1] | IgE to *Ascaris* spp [2] |
|---|---|---|---|---|---|---|
| ASC235 | M | 12 | ASA cohort | Asthma | 3.20 | 119.6 |
| ASC188 | F | 42 | ASA cohort | Asthma | 0.85 | 9.08 |
| ASC115 | F | 49 | ASA cohort | Asthma | 0.55 | 1.22 |
| ASC084 | M | 9 | ASA cohort | Asthma | 0.65 | 2.49 |
| ASC064 | M | 10 | ASA cohort | Asthma | 0.50 | 3.61 |
| ASC055 | M | 28 | ASA cohort | Asthma | 0.39 | 3.10 |
| ASC147 | F | 27 | ASA cohort | Asthma | 0.29 | 5.66 |
| ASC081 | F | 9 | ASA cohort | Asthma | 0.27 | 60.2 |

[1] OD: Optical density units. As determined by ELISA

[2] kU/l as determined by ImmunoCAP.

**Table S2** Peptide List of natural Asc l 5. Tryptic peptides coinciding with Asc l 5 sequence after nano-LC-MS/MS of the *A. lumbricoides* extract.

| Peptide | -10lgP | Mass | Length | ppm | m/z | RT | PTM |
|---|---|---|---|---|---|---|---|
| KDEEKTDPEIEADIDAFVAK | 103.00 | 22.620.903 | 20 | 1.1 | 11.320.537 | 84.50 | |
| DEEKTDPEIEADIDAFVAK | 102.93 | 21.339.954 | 19 | -0.5 | 10.680.044 | 102.04 | |
| VPPFLVGAPESAVKDFFELIKK | 88.26 | 24.303.562 | 22 | -1.3 | 6.085.955 | 147.46 | |
| Q(-17.03)TPSRVPPFLVGAPESAVKDFFELIK | 88.16 | 28.545.269 | 26 | -0.6 | 9.525.156 | 165.32 | Pyro-glu from Q |
| Q(-17.03)TPSRVPPFLVGAPESAVKDFFELIKK | 87.25 | 29.826.218 | 27 | -0.6 | 7.466.623 | 151.29 | Pyro-glu from Q |
| TDPEIEADIDAFVAK | 84.46 | 16.327.882 | 15 | -0.2 | 8.174.012 | 108.49 | |
| VPPFLVGAPESAVKDFFELIK | 80.50 | 23.022.612 | 21 | 0.0 | 7.684.277 | 167.40 | |
| DFFELIKKDEEKTDPEIEADIDAFVAK | 76.78 | 31.545.596 | 27 | 2.3 | 7.896.490 | 136.15 | |
| VPPFLVGAPESAVK | 72.46 | 14.097.917 | 14 | 0.1 | 7.059.032 | 86.62 | |
| PEIEADIDAFVAK | 68.23 | 14.167.136 | 13 | -0.2 | 7.093.640 | 96.59 | |
| IKETMESLPK | 55.06 | 11.746.267 | 10 | -1.0 | 5.883.201 | 29.24 | |
| LTAIAEDAKLNGIQK | 53.43 | 15.838.882 | 15 | -1.0 | 7.929.506 | 49.78 | |
| D(+226.08)EEKTDPEIEADIDAFVAK | 52.41 | 23.600.728 | 19 | -1.5 | 11.810.419 | 83.81 | Biotinylation |
| EEKTDPEIEADIDAFVAK | 51.93 | 20.189.684 | 18 | -2.2 | 6.739.952 | 94.30 | |
| DFFELIKKDEEK | 51.27 | 15.397.820 | 12 | -1.4 | 7.708.972 | 72.54 | |
| IKETM(+15.99)ESLPK | 50.40 | 11.906.217 | 10 | -0.5 | 5.963.178 | 28.79 | Oxidation (M) |
| DFFELIKK | 48.67 | 10.385.750 | 8 | 0.4 | 5.202.950 | 78.50 | |
| LTAIAEDAKLNGIQKR | 48.55 | 17.399.894 | 16 | -0.2 | 5.810.036 | 36.85 | |
| LTAIAEDAK | 48.18 | 9.305.022 | 9 | -0.6 | 4.662.581 | 29.93 | |
| EVRDELEKAIAGGA | 43.40 | 14.567.521 | 14 | 1.3 | 7.293.843 | 67.37 | |
| DFFELIK | 42.43 | 9.104.800 | 7 | -0.7 | 4.562.469 | 105.59 | |
| IKETMESLPKEVRDELEK | 42.29 | 21.731.299 | 18 | -0.9 | 5.442.892 | 64.63 | |
| EVRDELEK | 42.19 | 10.165.138 | 8 | -0.4 | 5.092.640 | 25.88 | |
| AHEAEYEK | 41.48 | 9.754.297 | 8 | -0.3 | 4.887.220 | 24.37 | |
| AHAAAIAK | 37.29 | 7.514.340 | 8 | -0.8 | 3.767.240 | 27.50 | |
| KDEEKTDPEIQ(sub E)ADIDAFVAK | 36.41 | 22.611.062 | 20 | 3.0 | 5.662.855 | 92.13 | |
| **total 26 peptides** | | | | | | | |

**Figure S1** SDS-PAGE of samples used for Western blot probed with sera pool from individuals with positive IgE to *Ascaris*.

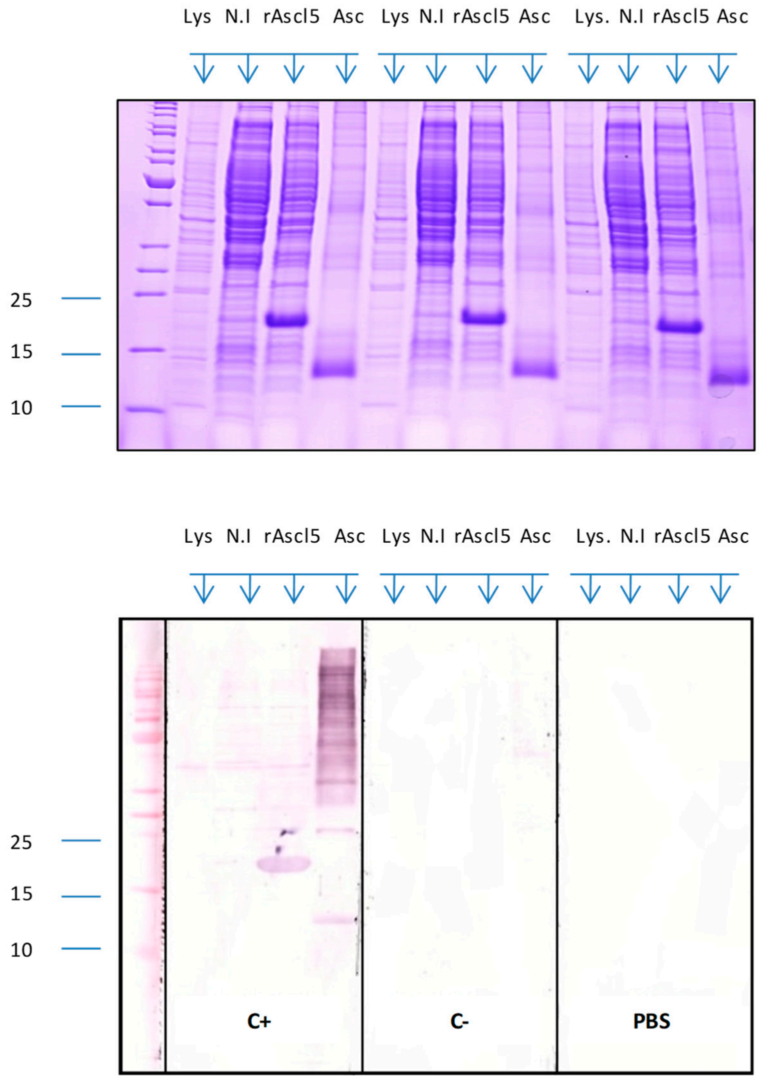


**Supplementary Figure 1.** Lys) *E. coli* Origami (DE3) lysate, N.I) non-induced transformed culture lysate of *E. coli* Origami (DE3), rAsc l 5) induced transformed culture lysate of *E. coli* Origami (DE3) and Asc) *Ascaris lumbricoides* extract.

C+ (Sera pool with positive IgE to *Ascaris*); C- (Sera pool with negative IgE to *Ascaris*); PBS buffer control.

**Figure S2** SDS-PAGE of rAsc l 5

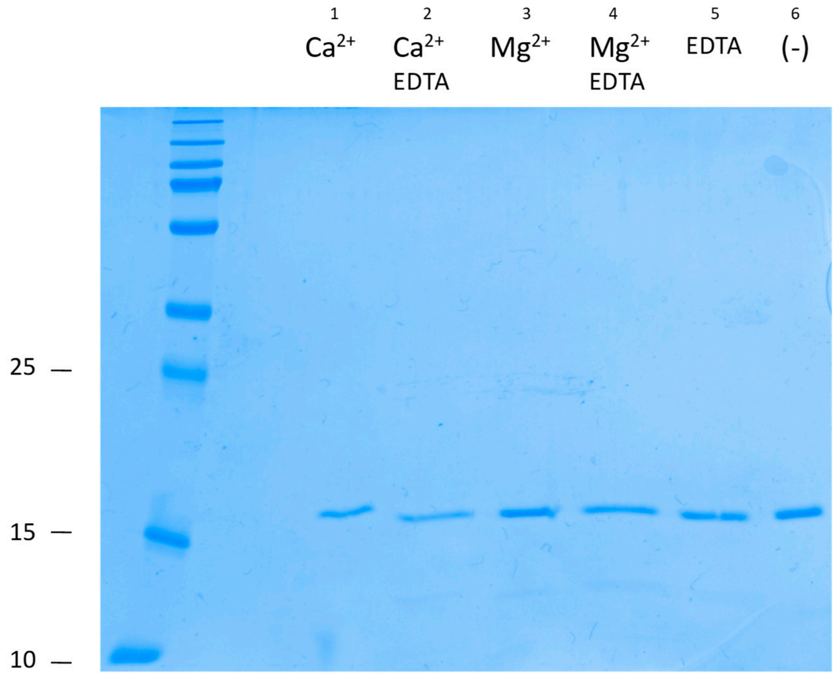


**Supplementary Figure 2.** SDS-PAGE. Same samples from CD experiments were used for in lanes 1, 3 and 6. EDTA was added to the preparations as a control (lanes 2, 4 and 5). Concentrations were as follow: 100 μg/mL rAsc l 5, 74 mM $CaCl_2$, 221 mM $MgCl_2$, or 250 mM EDTA as needed. 1) rAsc l 5 + $CaCl_2$; 2) rAsc l 5 + $CaCl_2$ + EDTA; 3) rAsc l 5 + $MgCl_2$; 4) rAsc l 5 + $MgCl_2$ + EDTA; 5) rAsc l 5 + EDTA; 6) rAsc l 5 alone. Sample volume 12 μL.

**Figure S3** Calcium and magnesium binding sites in Asc l 5

**A**

SXP motif 1

26 29 33 37

QTPSRVPPFLVGAPESAVKDFFELIKKDEEKTDPEIEADIDAFVAKLGA 49

SXP motif 2

66 70 73 88

DYTNKFIAFKAELKAHEAEYEKAHAAAIAKFSPAAKEADAKLTAIAEDA 98

KLNGIQKRQKIKETMESLPKEVRDELEKAIAGGA 132

**B**

| **Predicted divalent cation binding sites in Asc l 5** | | |
|---|---|---|
| **$Ca^{2+}$ binding residues** | **1)** | **26K, 29E** |
| | **2)** | **33D, 37E** |
| | **3)** | **66E, 70E** |
| **$Mg^{2+}$ binding residues** | **4)** | **70E, 73H, 88D** |

**C**

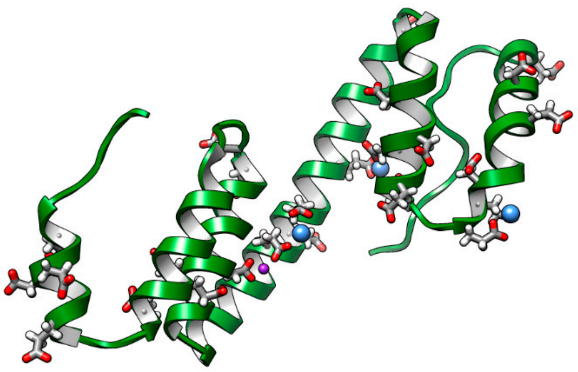

**Supplementary Figure 3.** Predicted binding sites in Asc l 5. S1A: In mature sequence of Asc l 5 (MN275230) Asp and Glu residues are in red. Predicted calcium or magnesium binding residues are numbered from 26 to 88. S1B: List of residues predicted to bind calcium or magnesium. S1C: Asc l 5 model with calcium (blue) and magnesium (magenta) ions.
